# A qualitative exploration of the physical and psychological wellbeing of family carers of veterans in Australia

**DOI:** 10.1371/journal.pone.0269012

**Published:** 2022-06-03

**Authors:** Dannielle Post, Alison Barrett, Amy Baker, Jocelyn Kernot, Gaynor Parfitt

**Affiliations:** 1 Alliance for Research in Exercise, Nutrition, and Activity (ARENA), Allied Health and Human Performance, University of South Australia, Adelaide, South Australia, Australia; 2 Allied Health and Human Performance, University of South Australia, Adelaide, South Australia, Australia; University of Birmingham, UNITED KINGDOM

## Abstract

Family carers of veterans have a tendency not to seek support for their own wellbeing concerns. Understanding the barriers and enablers that family carers face in attending to their own wellbeing and in their caring role generally, is key to supporting family carers of veterans. This qualitative study sought to explore family carers’ experiences and perceptions of their caring role, using semi-structured interviews. Questions were designed to capture concepts related to the barriers and enablers family carers face in attending to their own wellbeing. Twenty-two family carers participated in interviews. Thematic analysis facilitated the identification of key themes including the impact of the caring role; a perceived lack of recognition or appreciation of the caring role; expressed preferences for support; and consideration of the family unit. Findings suggest a need for accessible and multi-faceted support services for family carers of veterans, that target the drivers of physical and psychological wellbeing.

## Introduction

There are approximately 2.8 million family carers in Australia, who in 2020 provided over 77 billion dollars’ worth of care in the community [1]. Family carers regularly put the needs of the people who they care for ahead of their own needs, and have a tendency to not seek support for their own physical and psychological health concerns [2]. This can result in family carers having a higher chance of illness and mental health issues [3]. Family carers of veterans face similar demands as those faced by other family carers, such as the need to provide assistance with activities of daily living or advocating on behalf of the care-recipient; however, their circumstances are often exacerbated by the unique nature of the veteran experience and Defence environment. Veterans may be physically incapacitated or be living with mental health issues, including post-traumatic stress or moral injury, and the family carer balances ‘normal’ family roles and responsibilities, including child care, managing the household, and maintaining employment, with their caring responsibilities. This may leave the family carer’s own needs unattended [4] and may result in compassion fatigue [5]. With recent focus on the escalating suicide rate of veterans, the appointment of a National Commissioner for Defence and Veteran Suicide Prevention, and the announcement of a Royal Commission into veteran suicide in Australia, it is important that the physical and psychological wellbeing of family carers of veterans, such as partners, parents, and adult children, is also supported.

There is substantial evidence to indicate that psychological and physical wellbeing are negatively impacted by leading a sedentary lifestyle, and having poor sleep quality [6, 7]. Physical activity is recognised to independently contribute to better physical and psychological wellbeing and to decrease the risk of developing chronic diseases, as well as benefiting mood, cardiovascular fitness and sleep [8]. Physical activity has also been recommended as an approach to preventing co-morbidities, such as cardiovascular disease, type 2 diabetes, and psychological conditions [9, 10] and for people with some types of cancer [11]. As such, understanding the physical activity behaviours of family carers and how they relate to wellbeing offers a potential pathway for interventions which support the wellbeing of family carers.

The qualitative analysis reported here is part of a larger, mixed-methods pilot study. The first phase of this mixed methods study, a quantitative analysis of the physical and psychological wellbeing of family carers of veterans, has been reported elsewhere [12]. Briefly, the findings indicated that family carers of veterans had higher levels of psychological distress and lower mental wellbeing compared to population norms [12], although resilience levels were considered normal-to-high. Family carers with higher scores for resilience demonstrated greater physical fitness, assessed by the six-minute walk test, and as resilience increased, the time spent in sedentary activity bouts decreased. Only 40% of family carers met the recommended 150 minutes physical activity per week [13]. Family carers with higher psychological distress slept longer. This quantitative analysis of the relationship between physical and psychological wellbeing factors for family carers is believed to be the first of its kind in a cohort of Australian family carers of veterans.

To contextualise the quantitative findings and to understand the barriers and enablers faced by family carers in meeting their own physical and psychological wellbeing needs, qualitative methods were used in the second phase of the study. Previous qualitative research exploring the experiences of family carers is often linked specifically to intimate partners of veterans with post-traumatic stress, rather than veterans generally. This research identified that family carers of veterans felt there was little understanding of their role, that they were not included in the care of their partner when it came to working with health providers, and that there was a need for peer support [14, 15]. These factors were reported to contribute to a sense of disconnect for carers, with such feelings potentially manifesting as stress and being barriers to coping in their caring role. Other research suggests that family carers of veterans feel isolated, with a sense of inequality in the relationship with their partner, and a loss of identity [16].

Through this research, we sought to understand the barriers and enablers that a cohort of Australian family carers of veterans face in attending to their own physical and psychological wellbeing as well as their experiences and perceptions of their caring role. This approach was intended to facilitate an accurate representation of the lived experience of family carers.

The key research questions to be addressed in the qualitative exploration of family carers’ experiences were:

What are the barriers family carers of veterans face in attending to their own physical and psychological wellbeing needs?
What are the enablers for family carers of veterans to attend to their own physical and psychological wellbeing needs?
Are family carers physically active?
How do family carers perceive their role?

## Methods

This research applies a qualitative descriptive approach [17], that is intended to facilitate an accurate representation of the lived experiences of family carers. This article reports the findings in accordance with *Consolidated Criteria for Reporting Qualitative Research* (COREQ) guidelines [18] (please see S1 Checklist COREQ), although we have not sought to quantify or assume data saturation, in agreement with recent discussions by Braun & Clarke [19] about concepts related to data saturation and sample size in the context of reflexive thematic analysis, whereby it is not possible to know ‘how many’ data items or participants are required, while planning the study. Further, while we are comfortable that we have addressed relevant concepts raised by family carers in these interviews, due to the very unique experiences of participants and the person they are caring for it is unlikely that this is an exhaustive representation of this topic. For clarity, further reference to carers in this article relates to family members of veterans, who provide daily support to veterans.

### Semi-structured interviews

Semi-structured interviews were chosen for the second phase of this study. Interview questions were designed by, and piloted among, the project’s stakeholder group, comprised of the research team, a lived experience family carer, and a veteran. Questions were designed to capture information related to the barriers and enablers that family carers faced in attending to their own wellbeing, and family carers’ perceived needs in undertaking their role, as well as questions related to carers’ current levels of physical activity and health behaviours. Using a co-design approach to interview question development, stakeholders’ knowledge and lived-experience perspectives helped shape the qualitative interview topic guide to ensure appropriate language and terminology was employed. The interview questions were piloted with the stakeholder group and no subsequent changes were required to the core questions. Please see Interview Guide in S1 File.

Interviews followed an iterative approach, where new avenues for discussion raised by individual family carers were explored during the interviews, encouraging them to expand on responses where relevant; and to incorporate concepts raised by other participants in prior interviews. Face-to-face interviews were favoured, in order to build rapport with participants, to help them to feel comfortable in the interview situation and enable assessment of body language and facial expressions. In situations where a face-to-face interview was not possible, the interview was completed over the phone. In this case, the researcher built rapport with the participant through casual conversation prior to the interview. Field notes related to specific points of interest were made during the interviews. Participants who indicated distress during the interview were recommended to contact their general practitioner and directed to relevant mental health support services. In cases where participants indicated that they would like to be involved in carer support groups, relevant contact details were provided to the participant.

Interviews were undertaken by a female research assistant, who at the time was undertaking a Master’s degree and had more than five years’ experience interviewing research participants, or the lead researcher, a female, PhD-trained researcher with more than ten years’ experience interviewing research participants. While both researchers had family members who had served in the armed forces, neither was a family carer for a veteran, nor had experienced living with a veteran in the period following their return from deployment. The interviewers debriefed after each interview, due to the nature of some of the information shared by participants.

### Recruitment and interview process

Participants were recruited for the quantitative and qualitative phases of the study between February and July 2019, with support from stakeholders involved with carers of veterans including The Road Home, Partners of Veterans South Australia, and Legacy Club of Adelaide and Broken Hill (Australia). Recruitment material included flyers, social media, word of mouth, and radio and television media. Snowball sampling occurred, with participants recommending participation in the study to other family carers in their networks. While some participants were known to the researchers as a result of associating with the same networks in the family carer space, these relationships were not personal in nature and there was no coercion or expectation that family carers would be involved in the research due to any perceived relationship with the researchers.

Family carers who were interested in participating in the project were invited to contact the research team by telephone or email, whereupon the researcher discussed the project with them, and they were subsequently sent the participant information sheet and consent form. Once the family carer advised they would like to participate, a time was scheduled to attend the university campus for the laboratory session (phase 1). Upon arrival, informed written consent to participate in the project was provided. In most cases, the qualitative interviews happened on the same day as the laboratory session, with only the interviewer and participant present. Upon completion of the interview, participants were provided with a $30 gift voucher in acknowledgement of their time.

### Analysis

Interviews were audio recorded, transcribed verbatim and uploaded to NVivo software program (QSR International Pty Ltd 2017) which facilitated development and organisation of the codes. Thematic analysis [20] was applied to identify higher order themes and sub-themes. An inductive approach was used [21] to understand and represent the experiences of participants; however, there were elements of a deductive approach, as we sought to identify themes that specifically related to the barriers and enablers that family carers of veterans face in attending to their own physical and psychological wellbeing, as well as their experiences and perceptions of their caring role. To become familiar with the data, initial thoughts were discussed between members of the research team during debrief sessions after each interview, and transcripts were read multiple times by the same researchers who undertook and transcribed the interviews. Sections of the content were coded based on their meaning and a second coder confirmed aspects of the coding, raising other codes for consideration. Codes were then categorised and candidate themes identified. These themes were subsequently refined, with any disagreements discussed with other members of the research team until consensus was reached [20]. The project’s stakeholder group, including lived-experience stakeholders, was involved in the sense-making of the data during the final write up of the research. This report was shared with all participants, a number whom reported back that they felt their experiences were suitably described. This approach was intended to make sure that our interpretations were truly reflective of the experiences of family carers. (Please see Audit trail example in S2 File for an example of the analysis.) This study was approved by the University of South Australia’s Human Research Ethics Committee (Protocol number 201648).

## Findings

### Participants

A total of 22 interviews were undertaken with family carers: 18 face-to-face and four over the phone. Thirteen participants cared for Vietnam Veterans and nine for veterans of more recent conflicts. The mean age of interviewees was 59.9 years (SD 13.7); 95.5% of interviewees identified as female (Please see Table 1 for interviewees’ demographic characteristics; data in S1 Dataset). Interviews took between 26 and 75 minutes, depending on the content raised and the level of elaboration by the participant.

**Table 1:** Demographic characteristics of interview participants (n = 22).

Characteristic	Mean (SD), % or range
Age	59.9 (13.7)
Gender (identified as Female)	95.5
Length of time in caring role (years)	2–50
Education	
Below year 12	13.6
Year 12 or equivalent	18.2
Vocational qualification	31.9
Associate/Advanced diploma	18.1
Bachelor’s degree	12.5
Master’s degree	4.5
Relationship to person being cared for	
Partner/spouse	95.5
Child	4.5

### Themes

There were a number of higher order themes identified that appeared to have an influence on family carers’ role, their capacity to attend to their own physical, social and emotional needs and their sense of wellbeing. These themes included: barriers to attending to own physical and psychological needs; enablers for attending to own physical and psychological needs; the impact of the caring role; a perceived lack of recognition or appreciation of the caring role; expressed preferences for support; and consideration of the family unit. The themes and their associated sub-themes are provided in Table 2 and explored in detail in subsequent sections. All names are pseudonyms to protect the identity of the family carer.

**Table 2:** Higher order themes and sub themes.

Higher order theme	Sub-themes
Barriers to attending to own physical and psychological wellbeing needs	Motivation and time
	Own health concerns and issues
	Not aware of service availability
	Lack of childcare
Enablers for attending to own physical and psychological wellbeing needs	Taking time to do things for themselves
	Motivation
	Social and partner support
	Enjoyment of physical activity
Impact of the caring role	Compromises or adjustments made
	A sense of guilt
	Shielding others from the reality of the situation
	Ongoing trauma
	Psychological wellbeing of children
	Physical health behaviours beyond physical activity (sleep, nutrition)
	Identity
	Intimacy
	Ability to engage in paid employment
Lack of recognition or appreciation of the caring role	Government and veterans’ organisations, and person being cared for
	Financial independence and implications
Expressed preferences for support services and networks	Support networks
	Group activities, retreats and respite
Consideration of the family unit	Recognition of different types of families
	Family-oriented training or education and services
	Childcare options or services

Most family carers perceived their role to see caring as just being something that they did. While they felt their role was not well acknowledged and underappreciated, not all of them perceived their role as negative or burdensome on their life. For many, they ‘just got on with it’, as these accounts explain:

…generally, I think that carers, the broad application of the term, will put themselves last and that’s not because they’re particularly selfless, I think it’s because that’s what needs to happen to get the job done and the people that succeed at that role are very practical people, and those that don’t, don’t do it.

[Bailey]

I think if you asked me these questions 30 years ago, then you might have got a different answer than, than what you’ve got today. I think you just tend to get on with it, and then um it becomes the norm.

[Tyler]

…I think we’re in a good place. I’m very lucky and I do think that I’m fortunate, we’ve had our journey, so to speak, and we’ve come out the other side and we’re strong so, I’m lucky, I do think I’m lucky.

[Morgan]

#### Barriers to attending to own physical and psychological needs

When it came to identifying and exploring the factors that served as barriers to family carers attending to their own wellbeing needs, a number of family carers were cognisant of the need to maintain their wellbeing. Multiple barriers were discussed by family carers in attending to their own needs, and their physical and psychological wellbeing, and not all were necessarily directly related to their caring role. These barriers included “a lack of motivation and time”; “having health concerns” that meant they had challenges looking after themselves, being “unaware of service availability” and a “lack of available childcare”. Other family carers spoke about their caring role being a priority, suggesting that the demands of the role left them with little time for their own needs:

I wish there was time for more but you know my priority is to get home from work to do all the jobs that need to be done with the children and dinner because I don’t know if my husband will be a in a place that he can do that.

[Ash]

It seems to be that he’s becoming more and more reliant and more and more clingy to me, like, you know, ‘What time will you be home? When will you be back?’, all that sort of stuff? And that’s happening more and more, and I’ve got less and less time for myself.

[Charlie]

…you cannot lay aside your carer’s role. It doesn’t matter if you’re lying in the trauma unit at [the] hospital screaming your head off, and you are in so much pain and you can’t think straight. It doesn’t matter because you’re not unconscious [so] you are still responsible [for the veteran], and I still had to make phone calls and get [veteran] looked after while I’m in hospital. That’s the really hard part of the carer’s role.

[River]

Mental and physical “health issues of their own” were also described as barriers to family carers attending to their own needs and impacting their capacity to be involved in social activities or hobbies. It seemed too, in a few cases, that the behaviours of the veterans also influenced the family carers’ sense of motivation and mood:

I have issues with um, medication for me is a problem, because if my asthma gets bad, I’m on steroids for 6 weeks before I can get off them again and then often I’m only off them for a couple of weeks and then I’m back on them again, so steroids are hopeless for trying to control your weight. I will admit that if I’m really miserable, then I do eat the wrong things.

[Fabian]

…Main barrier? Oh, just depression I suppose. Depression would be the main barrier. You know, the get up and go, um it’s easy just to get up and have breakfast and get on the computer, um, when someone’s [the veteran] lying around the house, you know, still lying in bed.

[Alex]

When it came to physical activity specifically, family carers reported different reasons for not being active, including factors associated with their caring role or concern about leaving the veteran they were caring for on their own:

I think it’s a combination of both [the caring role] and it’s a combination of I’m very tired, and I lack the motivation to do it as well. Yeah. And I guess I’m concerned about leaving him home on his own as well.

[Charlie]

I don’t know if it’s because of that activity, caring activity, or if it’s my own state of mind towards physical exercise, sometimes I think I’m just too lazy to do it, I just can’t be bothered, yeah so, I can’t really relate it to one or the other.

[Jo]

Well, it’s been 20 years [as a carer] …so I think it’s been a slow run down of my activities. I mean, you know, I was playing basketball, walking, swimming, aqua aerobics, but sometimes it just gets too hard. Like, he’s still in bed now. You know, sometimes he won’t get up ‘til 1 o’clock…

[Alex]

There were practical and access barriers for family carers in attending to their own psychological wellbeing through the use of professional services. It was apparent that some carers were “not aware of the services available” to support them specifically:

I didn’t know until this lady rang me up to introduce themself as a liaison officer for me… When she found out I was in hospital, she talked to me about what organisations [were available], and ‘we’ll provide the taxi for you’. And, I didn’t know they did that until I had a problem. And I guess for me, that’s the thing. All these things [that we don’t know about].

[River]

For some family carers, a “lack of available childcare” was also identified as a barrier to participation in self-care:

So, I think when you say about self-care, it would probably be good if there was childcare, so you know, you have so you don’t have to worry about your kids when you go to the gym and stuff.

[Lane]

…so there is no baby-sitting option, there is no creche option, basically they offer equine therapy, we’ve looked into twice, couples’ equine therapy and they do it … just for the serving member, ex-serving member, and there’s no creche provided so there’s literally no way we can access that program.

[Ash]

In other cases, systemic barriers were reported to make it difficult for family carers to access support. For example, carers reported wanting to make an appointment with a veterans’ counselling service for assistance as a couple and being told that the veteran had to make the appointment.

#### Enablers for attending to own physical and psychological wellbeing needs

In identifying the enablers to attending to their own needs, family carers acknowledged that “taking time to do things for themselves” was important, because ‘*if you didn’t*, *you’d be a slave to yourself*’ [McKenzie], and that they were conscious of making the time for these activities:

I have a massage occasionally, I like to go to the pictures, if it doesn’t suit some people oh well, I’ll go on my own.

[McKenzie]

I want something for me, and this [social group] was my avenue, for me.

[Charlie]

I’ve got a couple of good friends one in particular…and we’ll go out for lunch or we’ll go to the markets on the weekend…I still meet my friends from work for dinner, but I’ve done that for a long time ‘cos [veteran] was away so much, so if I didn’t have a social life of my own, then I wouldn’t, I wouldn’t have done anything.

[Fabian]

Some family carers acknowledged that the “motivation” for them to care for themselves was that if they did not look after themselves, they did not know who would look after the veteran they were caring for:

Because if I don’t do what I want to and keep myself active and keep myself well, who’s gonna look after him? And…who’s gonna look after me if I don’t look after me?

[Lee]

I think if you try to stay healthy and fit that helps with everything like mentally and, and the physical demand on caring for someone else. I mean you gotta look after yourself as well as look after them, because you can go downhill pretty quick looking after a person if you don’t care for yourself.

[Deon]

Some family carers expressed that having “support from the veteran” to attend to their own needs and activities encouraged them to attend social gatherings or events:

…I’m not made to feel like I need to not go anywhere or that I have to stay home with [veteran], so [veteran] encourages me to go, [veteran] probably likes their own lonely time from time-to-time.

[Jo]

I go out to some Ladies’ Night just by myself, that might be a couple of times a month, and [veteran] is quite happy for me to go. And that sort of means a lot.

[Deon]

Many of the family carers reported being physically active, engaging in individual or structured physical activity including walking, swimming, water aerobics, rowing, or gym classes. It was not always articulated by the family carers that there was a concerted effort by them to be physically active, or that it was important to be physically active, even when directly asked about physical activity; however, an “enjoyment of physical activity” and its benefit was expressed. For some family carers, these activities were articulated as ‘me time’ or ‘doing something for myself’:

I usually walk my dog for a couple of hours a day and I do the rowing…twice a week…for somebody in their 70s, I do more exercise than most people…When I’m rowing, I’m just on top of the world.

[Tyler]

I’d like to do walking. I love walking, that’s what I would prefer to do.

[Charlie]

Physical activity for me and taking care of myself is taking the dogs for a really long run…and being out for about an hour and a half. And because it’s hilly, yeah, you really get in that sort of cardiovascular workout…Yeah, that’s me taking care of myself. Okay, then we’ll go for the long run. That’s my mindfulness time. So, I’m walking, I’m taking them out, but that’s my switch off.

[Sam]

#### Impact of the caring role

Family carers reported that they changed their own behaviours, making “compromises and adjustments” to accommodate the needs of the veteran for whom they were caring, some of which may impact the carer’s own health. This included having to ‘survey’ places they attended for incidents or events that might ‘trigger’ the veteran and cause them to react negatively to situations, and being vigilant in social and public settings:

So, you’re living on eggshells, you know, you’re sort of thinking, you know, we can’t go walk down [the] mall, he can’t cope with going to the footy, because he can’t cope with crowds. So, your life changes, and…you know, your life, you’re always living [with], you know, is this gonna upset him?

[Alex]

…and you’ve got to pick a time to go places, more so than actual avoiding [going places]. Sometimes it’s not the right time to go and do something, you’ll do it at a different time…Yeah, when he’s settled down.

[Lee]

In some cases, but it’s important to note, not all, family carers reported a “sense of guilt” in taking time to attend to their own needs or leaving the veteran at home on their own:

And my big thing is, it’s a wonderful opportunity to go and I do want to go sometime but you’re leaving your spouse at home who may or may not be in a good way.

[Ash]

It becomes a learned behaviour being a carer and that taking time out for yourself, you feel like well, I really must get going now because I’ve got stuff to do. And I think for people, even for somebody coming along and doing, even doing this [the research project], for them, it would be taking time out.

[Sam]

Family carers also described “shielding family and others from the reality of the situation”: ‘They know, but they don’t know the extent of it’ [Alex]. This related to family carers not sharing information about their day-to-day lives with other people or by being ‘peace-makers’ in their own homes:

I tried to keep it from them, keep everything sweet and lovely, perfect, the perfect marriage, the perfect household, um, appearances were everything and I tried to protect the kids. If he was angry at one of the kids for something, I would take the responsibility, ‘oh I told them to do that’ or ‘that was my fault’, so, um…the kids had no idea of how bad things were until I left and I think at times they are still finding out because I’m discussing it more with them.

[Taylor]

[Since partner passed] …I love driving in my gate and not going: ‘What am I going to find at home now?’ It was, it was like walking on eggshells and running through a minefield. And people don’t get to see that, and I think for a lot of veterans’ wives and carers of people with PTSD, if the person is really liked and you then say: ‘actually, they’re a bastard to live with…’

[Sam]

Some family carers described that they felt “ongoing trauma” and experienced mental health issues as a result of living with and caring for a veteran:

And, the women you know, it’s very renowned that a lot of the women have got secondary post-traumatic stress. I mean, I do, he [veteran] comes up behind me unexpectedly and I’ll go through the roof.

[Dale]

I still look over my shoulder, I still am fearful.

[Taylor]

There was also an observed impact on the “psychological wellbeing of children” of veterans. In this situation, family carers described how they believed that the manifestation of psychological issues in their adult children was as a result of growing up as a child of a veteran:

I think [child] always felt that he was never good enough for his father and couldn’t’ve [long pause] been the ‘real man’ that his father thought he should be [laughs], if that makes sense?

[Lee]

My son especially is traumatised. I mean he’s 45, but he is terrified that he’s going to be his father again.

[Taylor]

Family carers described the impact of caring on “physical health behaviours beyond physical activity”, including sleep and nutrition. Poor quality sleep was reported by a number of family carers; this was either due to having a partner who had nightmares or sleep disturbances of their own, or due to having had a stressful day and thinking about ways to avoid similar situations, which impacted some family carers’ ability to sleep:

So yeah, it does impact my sleep because I do some of those things for her and then I guess occasionally the stress of ‘ah that wasn’t a great day, how do we mitigate the flow on effects of that?’.

[Bailey]

I go to bed and I toss and I turn…my brain doesn’t switch off… and start tossing and turning and thinking and so forth. I don’t sleep well.

[Casey]

Well, when [veteran] wasn’t well my sleep was terrible just because his sleep was terrible, so very restless and lots of snoring almost like sleep apnea, so I was waking up ten, twelve times a night, so that was exhausting and that went on for more than 12 months.

[Morgan]

Nutrition was reported as being of good quality by some family carers, whereas others recognised that their eating behaviours needed improvement. In one case, a family carer described their nutrition being impacted for a period of time due to their financial status and the need to rely on the support of an ex-service persons’ organisation for vouchers to buy food. This in turn had an impact on the family carer’s wellbeing, through the need to justify their situation to the supporting organisations prior to getting assistance:

Nutrition, pretty good with food. Maybe if you talked to us 4 years ago, hugely different, we had no money…[veteran] couldn’t work 4 years ago, I was supporting him, we just had nothing, would be trying to buy food with only $5. Trying to access foods, having to fill in lots of forms, felt like we were lying [and] this was to a charity…

[Rory]

I probably don’t eat as healthy as I should, I know. You know, it’s easy to grab a biscuit or something…So yeah, yeah, I know that, I know I don’t eat properly.

[Charlie]

Concepts of ‘self’ and the impact of the caring role on “identity” were raised. One family carer, who had not long before experienced the death of the veteran they were caring for, clearly articulated their perception of the loss of identity that came with being a family carer and the need for family carers to consider their identity:

So, I think for other carers, it’s that getting your own identity back as well. Because often you just are a carer, and it’s like, if you aren’t a carer, what would you be?

[Sam]

This extended to a sense of nostalgia around what might have been, and the cross-over between the partner role and the caring role was also acknowledged, from both positive and negative viewpoints. A long-time family carer raised concerns about “intimacy” in the couple-relationship, and some family carers described feeling unsure about whether issues they had with the veteran they were caring for were ‘general marriage issues’ or enhanced by the caring role, a role that some saw as relentless:

It’s very hard to separate. Because you know, as a carer, if you weren’t married to them, you go home at four o’clock or whatever.

[Casey]

…and you almost become a parent to your caree. You know, I mean, it’s, they rely on you quite a bit.

[Tyler]

… um the lack of intimacy is quite a challenge for younger women. And it may be even for younger men who are married to, are partners of veterans as well. So, I don’t know about that side, but I do know that the women had mentioned it.

[Tyler]

[veteran] did have the night sweats and even now, we sleep in different beds and, which is not nice. It’s nice to be able to sleep with your husband/partner.

[Alex]

Some family carers advised that their “caring role impacted their ability to engage in paid employment” and they had to change their working practices, for example to work from home or take casual work, or retire from the workforce earlier than intended, to ensure they were available to care for the veteran at times when needed. One family carer reported being fired for taking too much time off during a six-week period when the veteran they were caring for was in hospital for psychiatric care:

…about 10 years ago, 10, 15 years ago when [veteran] started to decline some more, I had to bring my [work] home…after two or three years it was halftime and then another two or three years, it was down to a few clients a week because my caring role couldn’t sustain it.

[River]

He was ringing me up every day [at work] and one day, what really made me decide [to retire], one day he rang me five times just to see if I was okay, was I gonna be home on time? And you never, if you’re going out, you never say I’ll be home at four o’clock. Because, if you’re not there, they panic. Yeah. And so, I always had an hour and a half, maybe two hours to I think whether I’m gonna be home and then if I’m home early, that’s fine.

[Casey]

It got to a point because of [veteran’s] medical that I applied for a lower paying job so I could be closer to home so if anything happened, I could duck out of work.

[Jo]

#### Lack of recognition or appreciation for the caring role

Family carers expressed frustration that their role as a family carer was not recognised or acknowledged as they felt it should be, and that there was limited understanding about the impacts of caring for a veteran living with or without post-traumatic stress disorder [PTSD] or other mental health conditions. This lack of recognition was perceived to come “from Government and veterans’ organisations”, as well as “from the veterans themselves”, and the general population:

…everything impacts around them all the time, your concerns, your wants, or needs aren’t really considered, and they’re not considered by DVA [Department of Veterans Affairs] either really, to some extent. I think that might be changing, but a bit too late for us.

[Tyler]

…Some of the wives went along to swimming with their husbands, but the husbands, because they get a Gold Card, get all freebies and the wives still are an appendage…if they have a veterans’ physical activity group [where] the wives were included as veterans, as equal to the veterans, I think that would make a difference. [Taylor] *A Gold Card is a treatment card that provides veterans with clinically required treatment for all medical conditions, as well as access to a range of services and support

(DVA 2022).

A family carer-parent described that there was a perception of others that family carers were seeking a benefit or hand-out for being a family carer, when instead they were simply seeking help:

… some of them asked me, ‘What do you want?’ And I was really quite shocked, because I hadn’t thought about that. And I think they were thinking that I was looking for some sort of benefit, or some sort of medical card or something, that I didn’t know. And I, I had to actually say to them, ‘you know, it’s not about a handout, it’s about a hand up.’

[Luca]

Some of the family carers conveyed there was “financial independence and implications” associated with their caring role, and that funding and support was tied to the veteran. For example, items such as car parking costs and the veteran’s Gold Card are not available for the family carer. And whilst acknowledging that the veteran deserves these for their service, the family carers identify the limited funding for them as a financial burden. This may also link to perceptions of a lack of recognition for their role as a family carer:

You know, parking’s expensive. But if like with the [counselling service], if the husbands come, they can get a car parking permit. You know, whereas we can’t use that. That’s just for them. And you know, that’s like that with a lot of things. They’re bringing in the new veterans’ card. Everything is for them. There’s nothing for us. All we can have is counselling. There’s no financial support. I’ve still got to have my own private health cover. If I want it. And there’s no Gold Card for me for medical. Whereas he’s got a Gold Card. You know, I don’t dispute the fact that they’ve earned it.

[Casey]

My biggest thing is, I know, I know partners won’t get a Gold Card until your partner dies—we don’t want that to happen either [laughs]. But my main thing is when, when your partner’s in hospital you know the, the parking and the traveling with the fuel can be a huge, huge thing.

[Deon]

One family carer suggested that financial status was a reason that some family carers may have stayed in a relationship with the veteran, particularly for Vietnam-era families, describing how females in that era generally stayed at home and raised children, and remained reliant on the veteran to provide financially for them as they aged:

Most of them don’t have their own income. They have the carers’ allowance or some of them might have the carers’ pension…but their financial needs… it’s tied up with what their husband has got. [Even] if they’ve been working women and have super, I think they still don’t feel as though they’re financially stable enough to find a new home and so on and walking out on the marriage that’s slowly killing them…

[Taylor]

#### Expressed preferences for support services and networks

Family carers highlighted the importance of having a “support network” or people to share their experience with; although differences in preferences for who comprised these support networks were noticed. In some cases, family carers indicated that they liked to be able to talk or to socialise with other family carers of veterans; whereas others wanted to be able to talk to people who had no association with the veteran community or that did not know the person that they were caring for. The comments of some family carers indicated that they were not aware of the supports already available to them:

I think having groups or networks for partners and carers to be able to come together and talk about what they are going through, a carers’ helpline [would be helpful].

[Jesse]

I think carers in our situation of veterans is very lonely. The loneliness is, even though you’ve got friends who are all in the same boat, there are times when you don’t want to hear their problem. You got enough of your own…but there’s nobody else. Do you know what I mean?

[Casey]

Who would I call if I needed help just to say like ‘…this is really hard, it’s been a hard day, week, month whatever’…I would not even know where to contact.

[Bailey]

I find it quite tricky. All the services, there are services out there, but often you don’t know what they are. You don’t know how to access them.

[River]

Some family carers spoke about “group activities and retreats” that had previously been available for family carers, and the benefit of such activities, lamenting that these had ceased when funding had run out. While there was acknowledgement that some veterans would be resistant to “respite” care, some of the family carers thought that this would be helpful for the family carer and the veteran:

For carers, respite is probably one of the big issues…and I know that a lot of veterans are reluctant to go into respite.

[Tyler]

#### Consideration of the family unit

A family carer-parent spoke about the concepts of family when it came to recognising family carers of veterans, raising the point that it is not just the ‘typical’ nuclear family that plays a role in the care of veterans, and that there are “different types of family”:

…I had approached a couple of Defence-related organisations that they, you know, have the word family in them. But actually, when you sort of contact them, or knock on the door, so to speak, and you’re a Mum, there’s actually no…you know, it’s only wives and partners and children. They don’t include that in their narrative. They don’t include parents. They don’t include mothers or grandparents.

[Luca]

Focusing on the family, including greater consideration of the family by Government organisations and through the provision of “family-oriented education and services”, was also considered important by family carers. This extended to providing education about PTSD specifically & information about how best to speak to children about the issues their veteran may be facing. The need for readily available “childcare options/services” in times of need was raised, particularly for families separated from extended family due to relocation and who had no other support to rely on:

So, my big thing is that we need more training and workshops for families because you know you’re thrown in the deep end, you know how do you deal with this, how do you, you know, care for them, how do you provide, find your own network?

[Ash]

I think DVA could be a bit more family friendly, um you know not knowing who else to contact at the time that things had hit rock bottom and being told to ring back in 3 months and my quick response was ‘well my husband will be dead in a month’ and their response was just ‘aww I’ll put you on hold’ kind of a thing and then go find something and that’s when they said to go to the [other service].

[Morgan]

…a part of that psycho-education is, how do you deal with the kids? How do you explain it [PTSD] to your kids? What’s good for your kids? What’s not? All that kind of stuff?

[Jesse]

…if you’re going to support a family…there needs to be a crèche attached to it you know…because we are different families, we don’t have a support network, we literally moved every 1 to 3 years.

[Ash]

## Discussion

The aim of this qualitative research was to explore and understand the experiences of family carers of veterans, and how these experiences impact the physical and psychological wellbeing of family carers. The quantitative findings, reported elsewhere [12], describe a population with higher levels of psychological distress and lower mental wellbeing compared to population norms [22, 23], although resilience levels were considered normal-to-high. Family carers with higher resilience were demonstrated to have greater fitness capacity. The psychological impact of service for partners and children of veterans has been reported in other research, and most recently summarised in a systematic review by Armour et al. [24].

The qualitative findings in our study provide context to the quantitative physical and psychological wellbeing measures collected in phase one of the study and identify a number of barriers that family carers face, and enablers that support family carers, in attending to their own needs. Four other higher order themes were identified: Impact of the caring role, Lack of recognition or appreciation of the caring role, Expressed preferences for support services and networks, and Consideration of the family unit.

Despite the number of family carers in the community and the many hours of care they provide, as of 2018, only 896,000 of these family carers received either a Carer Payment or Carer Allowance from the Australian Federal Government [25]. The economic role family carers play, by providing care around the clock, is not lost on the family carers themselves, some of whom mentioned ‘saving the government money’ by providing this care. Family carers acknowledged the veterans ‘deserved’ the benefits they received from the Government but felt that they too should receive greater financial support. Finances and generational influences of the Vietnam War era were raised by one family carer as the reason they believed some family carers stayed in their relationships. Such generational influences included suggestions that females in that era generally stayed home and raised children and may not have worked in paid employment outside of the home, meaning they received no superannuation, and they remained reliant on the veteran to provide for them. As reflected in this study, in other cases, family carers who did work may have reduced their work hours or retired as their caring role became more demanding, potentially impacting their financial status.

Family carers perceive that there is a lack of recognition of their role and contribution to the care of veterans, from governments, veteran-specific organisations, and sometimes even the veteran for whom they are caring. Of interest to the interviewers was how this perception may have shaped the lack of recognition that the family carers gave themselves. Often, when the family carer was asked about attending to their own needs, within a sentence or two of beginning their response, their attention and conversation was back on the veteran. When this became obvious after a number of interviews, the interviewer raised this with one of the participants, who laughed and said: ‘*yep*, *because that’s what you’re used to doing*’ [Sam]. These factors seem to solidify the role of the family carer, how they view themselves, and perhaps explain why their physical and psychological wellbeing is often neglected.

The findings related to family carers’ health behaviours and the influences of caring on physical activity provide avenues for further investigation as a means of improving health-related behaviours and outcomes. Family carers were aware of the requirement to attend to their own wellbeing needs, even if this awareness was voiced from the perspective of being able to continue caring for the veteran. Family carers who were physically active expressed enjoyment of physical activity and articulated time spent being active as time for themselves. Findings of this qualitative analysis and the factors that relate to psychological wellbeing support the findings of other qualitative investigations involving family carers of veterans, particularly around concepts associated with recognition of the caring role [15], and identity as a family carer [16]. We consider that formal recognition of the family caring role is likely to have a substantial impact on family carers’ sense of identity, self-worth, and self-esteem, all of which contribute positively to wellbeing [26].

There appears to be evidence of psychological trauma for some of the family carers of veterans who participated in this study, with findings of this qualitative analysis reflecting the high distress scores indicated in the quantitative analysis [12]. These qualitative findings are similar to recent research that highlighted a self-reported decline in the mental health of intimate partners of veterans in the United Kingdom [27]. Further, there are suggestions of intergenerational trauma, with some of the family carers reporting that their now adult children are living with psychological conditions that they attribute to growing up with a veteran. A recent Australian exploration of the experiences of adolescent children of veterans diagnosed with PTSD suggested that there was ‘intergenerational transference of silence and avoidance’ related to mental health [28]. This may translate to children of veterans not seeking support for their own mental wellbeing. Whether the impact on the children of the family carers in our study is directly associated with the individual veteran’s service or is a reflection of the level of mental ill health within the general population cannot be verified and should be investigated further. Regardless, previous research has demonstrated the impact of service on family members [24, 29, 30]. In dealing with this trauma, it would seem that family carers in this study have difficulty in accessing mental health services provided for veterans and their families, and that the costs associated with private mental health services as an alternative are prohibitive; an issue also faced by the wider community.

The altruistic involvement in the research of family carers from the Vietnam era was clear. A number perceived that it was too late for them, but that they wanted to be involved in the hope that they might contribute to improved conditions and outcomes for family carers of current-serving veterans, the veterans, and their families. Family carers voiced concern that nothing had seemed to change from the post-Vietnam days, and that despite their own involvement in research over the years, history seemed to be repeating itself insofar as the level of trauma displayed by some veterans and their families. As one family carer stated about being involved in this research: *‘I really*, *really wanted to get my story out there so that maybe someone else doesn’t have to go through this [expletive]’* [Taylor].

This research demonstrates an identified need for more visible, accessible, and structured support for family carers of veterans, supporting previous research that identified the need for support for partners of veterans living with PTSD, particularly with respect to increasing PTSD health literacy [31]. Our findings suggest that this includes education around the signs of PTSD, and how to discuss mental health conditions with children. The availability of such services should be extended to all informal carers of veterans, regardless of their relationship to the veteran. Embedding information about intimacy and associated support services within education for partner-carers may be beneficial. There is a need for programs and services that target and support the family unit, that do not seek to paint family carers as martyrs nor blame veterans as the cause of all of the issues that might be present in the family unit. Such programs and services should incorporate components that target the carer, veteran, and where applicable, children, individually and as a family unit.

## Limitations

The findings of this research are not generalisable to all family carers of veterans and are likely more representative of the experiences, and enablers and barriers to physical and psychological wellbeing for family carers in the Vietnam-era than they are for family carers of veterans of contemporary conflicts and service. The majority of the participants identified as female, with some in same-sex relationships; however, the perspective of family carers who identify as male is not as extensively represented. While this is reflective of the male-dominated Defence Forces of the Vietnam-era, it is not necessarily reflective of the current landscape, where females comprise approximately 17% of the Australian Defence Force [32]. Anecdotally, we are aware that some family carers chose not to participate in this research due to the physical assessments performed in the quantitative analysis of the project. This was related to concerns that they would be judged for not being as healthy as they could be. As such, the family carers who participated in the interviews may be more physically active or health literate than other family carers. Further, while one parent-carer was involved in the study, the perspectives of family carers beyond that of partners, such as parents, grandparents, siblings, and children, are not well represented and should be explored further.

## Future directions

The next phase of this research is to broaden the lens on family carers, incorporating the perspectives across the range of family carers to understand the drivers of physical and psychological wellbeing, and the emotional and practical supports required by family carers. Additional investigations should focus on identifying the supports that are required at the level of the family unit. Interventions that serve to improve physical and psychological wellbeing, through targeting the issues raised by family carers, should be developed, trialled, and evaluated for effectiveness. Programs designed to increase physical activity, build resilience, and reduce psychological distress may be beneficial for family carers; however, these interventions will need to be contextually specific, co-designed with family carers to accommodate their varied needs, and be accessible, irrespective of family carers’ location or financial status. Beyond this is the need to effectively translate research findings into meaningful and practical outcomes for family carers of veterans and the family unit. This requires effective engagement at the policy-level, and researchers and family carers working together to advocate for change. As raised by multiple family carers: *‘they’ve been asking us the same questions for 40 years and nothing has changed…’*

## Supporting information

S1 File Interview guide. (PDF)

S2 File Audit trail example. (PDF)

S1 Checklist COREQ. (PDF)

S1 Dataset Demographic data reported in Table 1. (SAV)
