# Supplementary material for: A qualitative exploration of the physical and psychological wellbeing of family carers of veterans in Australia
Source: PLoS One. 2022 Jun 3;17(6):e0269012. doi: 10.1371/journal.pone.0269012 (PMC9165811; doi:10.1371/journal.pone.0269012)
Supplement: S1 File — (PDF) [file pone.0269012.s001.pdf]

S1 File. Interview Guide

Interview Guide

Additional questions will be informed by individual participant's responses to the surveys.

Themes to be addressed during the interview:

Ask about their experience as a carer, for example, how long they've been caring for the returned service person – distinguish this from their caring role as a parent, child, partner etc.

Current physical activity levels/behaviours.

Ask carers about how much physical activity (PA) they currently do.

What type of activities they do.

How often are they physically active?

What do they consider as PA (i.e. is there just a focus on gym, rather than incidental PA?)?

Has their level of PA changed as a result of their caring duties?

If so, how?

Health behaviours

Ask about sleep behaviours, whether the interviewee sleeps well, whether the carer has broken sleep due to their caring role (i.e. attending to the person for whom they care during the night etc.)

Investigate other health behaviours, such as nutrition, drinking, smoking, other coping behaviours in relation to individual participant's previous responses to surveys.

Needs of carers

Explore the needs of carers, whether they are met or unmet.

Ask carers whether they are able to do the things they want to do – this may relate to a number of things, not just PA.

Ask whether carers make time for themselves

Ask about carers' feelings around the time (if any) they allow for their own activities (this is exploring feelings of guilt, frustration, isolation, loneliness, for example).

Ask carers about the barriers that they face in attending to their own physical health needs.

Ask carers about the barriers that they face in attending to their own mental health needs.

Ask carers about the barriers that they face in attending to their own physical activity needs.

Ask carers about the enablers in attending to their own physical health needs.

Ask carers about the enablers in attending to their own mental health needs.

Ask carers about the enablers in attending to their own physical activity needs.

Ask carers about what they want. Are there services they need that are not provided.

At the end of the interview, ask carers if there is anything additional they would like to add, points they would like to make, that have not been already covered in the interview.

Be sure to thank the carers for their time.
