# Supplementary material for: A qualitative exploration of the physical and psychological wellbeing of family carers of veterans in Australia
Source: PLoS One. 2022 Jun 3;17(6):e0269012. doi: 10.1371/journal.pone.0269012 (PMC9165811; doi:10.1371/journal.pone.0269012)
Supplement: S2 File — (PDF) [file pone.0269012.s002.pdf]

S3 File Audit trail example

Example of analysis for the theme: Barriers to attending to own physical and psychological needs

Theme	Barriers to attending to own physical and psychological wellbeing needs							
Sub-themes	Motivation and time		Own health concerns and issues		Not aware of service availability		Lack of childcare	
Categories	Lack of motivation	Not enough time for exercise & self-care	Carers' physical health concerns	Carers' mental health concerns	Services available for veteran but not the carer	Didn't know about services	Lack of family support	Can't access programs without a creche
Codes (examples)	Lack the motivation		Had several health issues		Not treated the same, they're [wives] lesser		Childcare	
	Just too lazy		Depression the main barrier		All these things we don't know about		No baby-sitting, no creche available	
	Wish there was time for more		A lot of pain		Would not even know where to contact		Support network	
